# Reflex Detection of Ciprofloxacin Resistance in Neisseria gonorrhoeae by Use of the SpeeDx ResistancePlus GC Assay

**DOI:** 10.1128/JCM.00089-21

**Published:** 2021-04-20

**Authors:** Darren Y. J. Lee, Melinda M. Ashcroft, Eric P. F. Chow, Michelle Sait, Vesna De Petra, Marlene Tschaepe, Sigrid Lange, George Taiaroa, Catriona S. Bradshaw, David M. Whiley, Christopher K. Fairley, Benjamin P. Howden, Marcus Y. Chen, Shivani Pasricha, Deborah A. Williamson

**Affiliations:** a Department of Microbiology and Immunology, The Peter Doherty Institute for Infection and Immunity, The University of Melbourne, Melbourne, Victoria, Australia; b Melbourne Sexual Health Centre, Alfred Health, Carlton, Victoria, Australia; c Central Clinical School, Monash University, Melbourne, Victoria, Australia; d Centre for Epidemiology and Biostatistics, Melbourne School of Population and Global Health, The University of Melbourne, Melbourne, Victoria, Australia; e Microbiological Diagnostic Unit Public Health Laboratory, The Peter Doherty Institute for Infection and Immunity, The University of Melbourne, Melbourne, Victoria, Australia; f The University of Queensland Centre for Clinical Research (UQ-CCR), Faculty of Medicine, The University of Queensland, Brisbane, Queensland, Australia; g Department of Microbiology, Royal Melbourne Hospital, Melbourne, Victoria, Australia; Medical College of Wisconsin

**Keywords:** *Neisseria gonorrhoeae*, molecular diagnostic, antimicrobial resistance, whole-genome sequencing

## Abstract

Resistance-guided therapy (RGT) for gonorrhea may reduce unnecessary use of broad-spectrum antibiotics. When reflexed from the Aptima Combo 2 assay, the ResistancePlus GC assay demonstrated 94.8% sensitivity and 100.0% specificity for Neisseria gonorrhoeae detection. Of the 379 concordant N. gonorrhoeae-positive samples, 86.8% were found to possess the *gyrA* S91F mutation, which was highly predictive for ciprofloxacin resistance and stable across 3,144 publicly available N. gonorrhoeae genomes. Our work supports the feasibility of implementing RGT for gonorrhea into routine molecular workflows.

## Abstract (precis)

Resistance-guided therapy (RGT) for gonorrhoea may reduce unnecessary use of broad-spectrum antibiotics. When reflexed from the Aptima Combo 2 assay, the ResistancePlus GC assay demonstrated 94.8% sensitivity and 100.0% specificity for Neisseria gonorrhoeae detection.

## INTRODUCTION

The increasing incidence of gonorrhea globally is a major public health threat. For decades, gonorrhea has been treated before antibiotic susceptibility results are known, according to local treatment guidelines that are generally based on the local prevalence of antimicrobial resistance (AMR). In many settings, dual empirical therapy with oral azithromycin (1 g) and intramuscular ceftriaxone (500 mg) is recommended (1). With recent reports of increasing resistance, individualized therapy based on the molecular detection of resistance determinants (resistance-guided therapy [RGT]) has been suggested as a way of improving antimicrobial stewardship and delaying the emergence of AMR (2, 3). In particular, RGT for ciprofloxacin has been incorporated into gonorrhea treatment guidelines in the United Kingdom and was shown to be feasible and effective in a recent multisite clinical study in the United States (4, 5).

Ciprofloxacin resistance in N. gonorrhoeae occurs predominantly through point mutations in the DNA gyrase A gene (*gyrA*), most commonly a single point mutation at the serine 91 codon (GyrA S91F), which is highly predictive of ciprofloxacin resistance. Other mutations associated with increased ciprofloxacin MICs include a point mutation at the aspartic acid 95 codon of *gyrA* (D95), and mutations in the topoisomerase IV *parC* gene (6). The highly recombinogenic nature of N. gonorrhoeae means that continuous surveillance is critical to ensure the ongoing utility of diagnostic markers used for RGT and to detect “diagnostic escape variants” (2). Accordingly, the aims of this study were: (i) to assess the performance characteristics of a recently introduced commercial assay for ciprofloxacin RGT against a widely used nucleic acid amplification test (NAAT) for N. gonorrhoeae; and (ii) to determine the genomic stability of molecular determinants of ciprofloxacin resistance in a large collection of publicly available N. gonorrhoeae genomes.

## MATERIALS AND METHODS

Clinical samples were obtained from the Melbourne Sexual Health Centre (MSHC), the largest public sexual health service in Melbourne, Australia. A total of 445 clinical samples were collected from March to May 2019 and were stored at room temperature in Hologic Aptima unisex specimen transport tubes (Hologic, San Diego, CA, USA) as per the manufacturer’s instructions and tested for Chlamydia trachomatis and N. gonorrhoeae using the Aptima Combo 2 assay (AC2; Hologic, San Diego, CA, USA) within 24 h. Where available, consecutive clinical samples were collected. In the AC2 assay, transcription-mediated amplification (TMA) of diagnostic targets results in the production of luminescent signals, quantified as relative light units (RLUs). The RLU value is then used to categorize results as positive, negative, and equivocal (7).

In total, 400 μl of remnant AC2 samples underwent DNA extraction using the QIASymphony DSP virus/pathogen midi kit complex 400 protocol, as per the manufacturer’s instructions (Qiagen, Hilden, Germany). These samples were stored as per the manufactures instructions at room temperature prior to testing on the Aptima assay, and were all tested within 14 days post-NAAT. PCR testing for N. gonorrhoeae and the GyrA S91F mutation was performed on 5 μl of extracted DNA using the previously described ResistancePlus GC assay (SpeeDx Pty Ltd., Sydney, Australia) (8, 9) on a LightCycler 480 II (LC480 II; Roche, Switzerland). Briefly, the assay reports detection across five channels using the following targets: (i) detection of the N. gonorrhoeae *opa* gene; (ii) detection of the N. gonorrhoeae *porA* gene; (iii) detection of *gyrA* S91 (wild type); (iv) detection of *gyrA* S91F; and (v) an internal control to monitor extraction efficiency and qPCR inhibition. Interpretation of the results was performed using the ResistancePlus GC (7500) analysis software. The assay reports the following results: (i) whether N. gonorrhoeae was detected or not detected, and (ii) if N. gonorrhoeae was detected, whether *gyrA* is wild type, a *gyrA* S91F mutation, or indeterminant. Statistical analyses were conducted using GraphPad Prism (version 8.4.3). Binomial 95% confidence intervals (CI) were calculated for all proportions. Differences between groups were calculated using either a Mann-Whitney test or a chi-square test. Bioinformatic analyses are described in the supplemental material.

### Ethical approval.

This study was approved by the South Eastern Sydney Local Health District Human Research Ethics Committee (HREC/17/POWH/510).

## RESULTS AND DISCUSSION

### Assessment of the ResistancePlus GC assay for detection of Neisseria gonorrhoeae.

In total, 445 clinical samples from different anatomical sites (from 336 patients) were tested using the ResistancePlus GC assay (400 N. gonorrhoeae NAAT-positive and 45 N. gonorrhoeae NAAT-negative Aptima samples) (Table 1). In total 97/336 (28.9%) patients had samples from ≥1 anatomical site. Compared to Aptima NAAT, the overall sensitivity and specificity of the ResistancePlus GC assay for detection of N. gonorrhoeae was 94.8% (379/400; 95% CI = 92.6% to 97.0%) and 100% (45/45; 95% CI = 97.8% to 100.0%), respectively. There was a significant difference in RLU values as reported by the AC2 assay between detected N. gonorrhoeae-positive samples (median RLU 1,536, interquartile range [IQR] 1,453 to 1,583 RLUs) on the ResistancePlus GC assay compared to undetected/discordant samples (median RLU 863, IQR 455 to 1,205 RLUs, *P < *0.001), which may suggest a lower bacterial load in negative samples.

**TABLE 1:** Specimen type for clinical samples tested using both Aptima Combo 2 and ResistancePlus GC molecular assays^*a*^

Site	Sample bank 1: N. gonorrhoeae-positive (no. [%])	Sample bank 2: N. gonorrhoeae-negative (no. [%])
Anorectal	150 (37.5)	15 (33.3)
Pharyngeal	176 (44.0)	22 (48.9)
Urogenital	74 (18.5)	8 (17.8)
Total number	400	45

a Values are given as number of samples (percentages). Sample bank 1 consisted of 400 N. gonorrhoeae NAAT-positive clinical samples that were previously stored for 14 days at room temperature in Hologic Aptima unisex specimen transport tubes (Hologic, San Diego, CA, USA) between 14 March and 29 May 2019. Sample bank 2 consisted of 45 N. gonorrhoeae NAAT-negative clinical samples collected from routine gonorrhea testing in September 2019.

### Identification of *gyrA* alleles.

**FIG 1:**
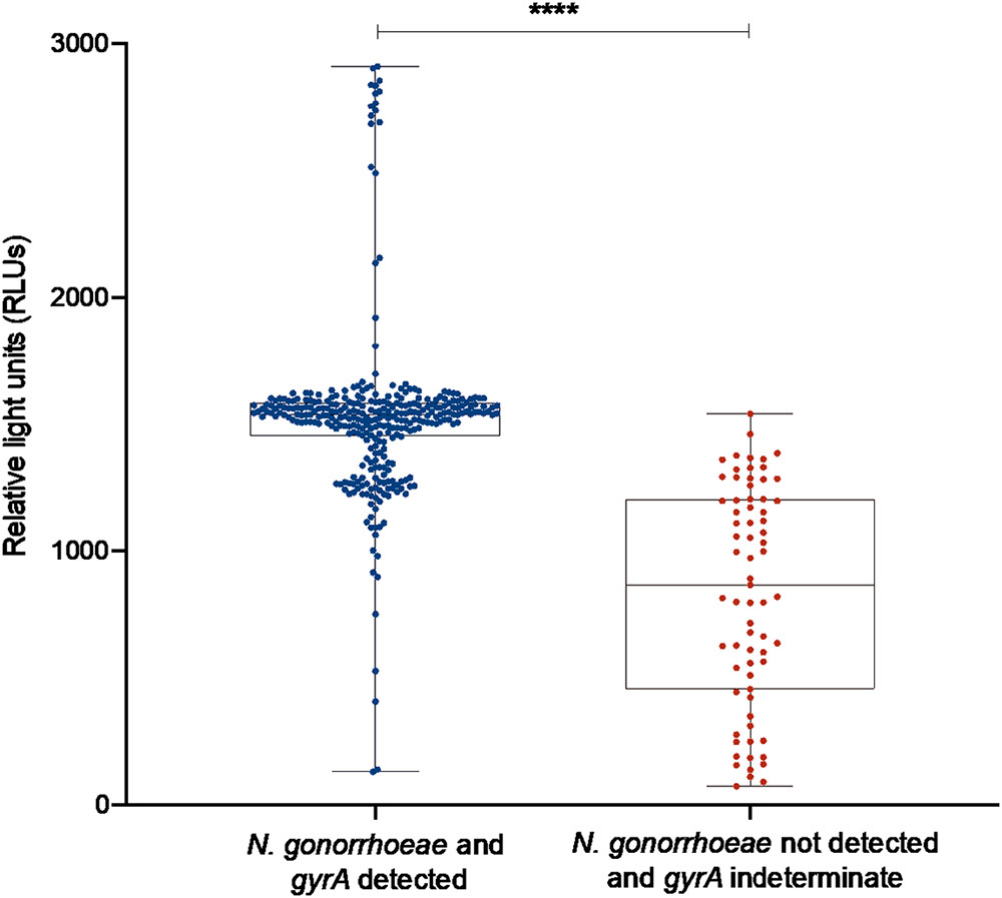
Detection of Neisseria gonorrhoeae and characterization of *gyrA* using the ResistancePlus GC assay in relation to relative light units (RLUs) reported by the Aptima Combo 2 assay. Boxes depict the interquartile range, and the median is represented by a short black line within the box. Whiskers represent the 5th and 95th percentiles and dots represent individual samples. Statistically significant differences between median RLUs are indicated with asterisks (****, *P < *0.0001).

**FIG 2:**
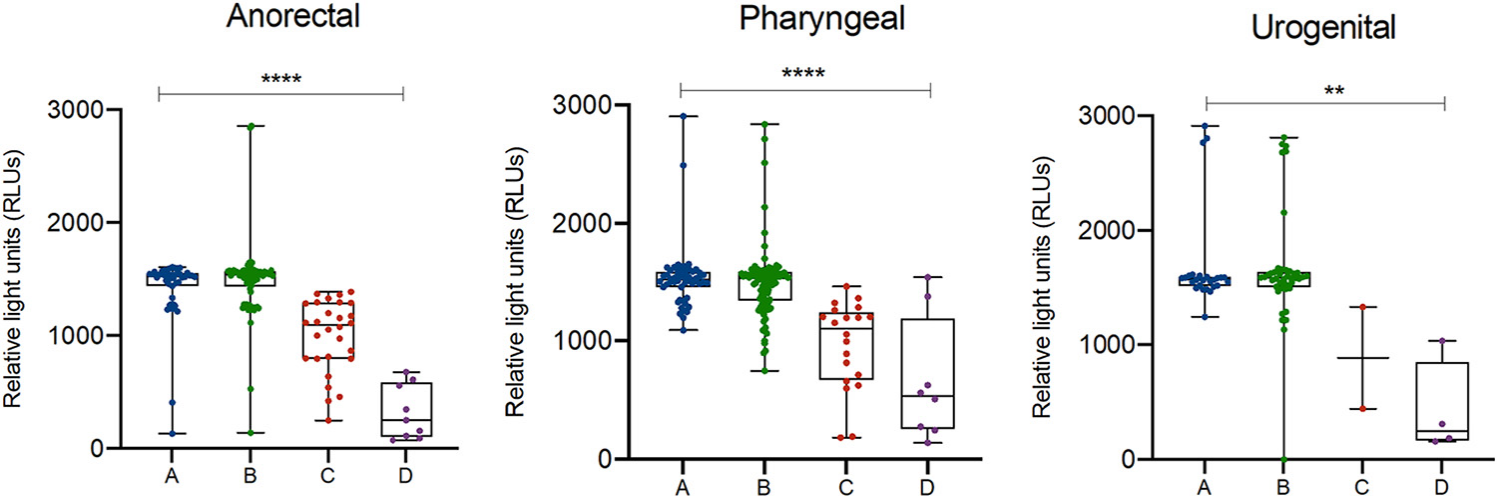
Detection of Neisseria gonorrhoeae and characterization of *gyrA* in samples derived from different anatomical sites of infection using the ResistancePlus GC assay. Boxes depict the interquartile range, and the median is represented by a short black line within the box. Whiskers represent the 5th and 95th percentiles and dots represent individual samples. Statistically significant differences between median RLUs are indicated with asterisks (****, *P < *0.0001; **, *P < *0.01). (A) N. gonorrhoeae detected and *gyrA* S91F mutant detected; (B) N. gonorrhoeae detected and *gyrA* S91 WT detected; (C) N. gonorrhoeae detected and *gyrA* indeterminate; (D) N. gonorrhoeae not detected.

The ResistancePlus GC assay successfully generated a *gyrA* result in 329 (86.8%) of 379 samples that were positive for N. gonorrhoeae by AC2 (Table 2). Of these, 206/329 (62.6%) samples had a *gyrA* S91 wild type (WT) result, and 123/329 (37.4%) had a *gyrA* S91F mutation. The remaining 50/329 (15.2%) N. gonorrhoeae-positive samples were indeterminate for *gyrA* (i.e., the ResistancePlus GC assay could not determine whether a WT or mutant *gyrA* was present). Given that the samples were found to be N. gonorrhoeae positive by the AC2 and ResistancePlus GC assays, it is unlikely the indeterminate result was due to cross-reactivity with nongonococcal strains. Instead, it is likely that the *gyrA* detection sensitivity is lower than N. gonorrhoeae detection. This is consistent with previous work by Cotton et al. 2020 that reported a sensitivity of 97.1% for detection of *gyrA* and a sensitivity of 98.5% for detection of N. gonorrhoeae (10). In our study, samples with indeterminate *gyrA* results had significantly lower AC2 reported RLU values compared to samples with *gyrA* detected (median RLU 1,091 versus 1,536, *P < *0.001) (Fig. 1; Table S1 in the supplemental material). Indeterminate *gyrA* results were significantly more likely in anorectal samples (28/150; 18.7%) and pharyngeal samples (20/176; 11.4%) compared to urogenital sites (2/74; 2.7%, *P < *0.001) (Fig. 2). A limitation of our study was the lack of associated phenotypic data; this limitation reflects the increasing use of molecular testing, which reduces the availability of isolates for additional analyses.

**TABLE 2:** ResistancePlus GC results for the detection of N. gonorrhoeae and *gyrA* in different anatomical sites of infection^*a*^

Site	No. (%) N. gonorrhoeae detected, *gyrA* indeterminate	No. (%) N. gonorrhoeae detected, *gyrA* mutation detected	No. (%) N. gonorrhoeae detected, *gyrA* mutation not detected	No. (%) N. gonorrhoeae not detected	No. total
Anorectal	28 (7.0)	43 (10.8)	70 (17.5)	9 (2.2)	150
Pharyngeal	20 (5.0)	54 (13.5)	94 (23.5)	8 (2.0)	176
Urogenital	2 (0.5)	26 (6.5)	42 (10.5)	4 (1.0)	74
Total number	50	123	206	21	400

a Values are given as number of samples (percentages). Results for the 400 clinical N. gonorrhoeae NAAT-positive samples as reported by SpeeDx ResistancePlus GC assay.

### Genomic assessment of *gyrA* S91F across Neisseria gonorrhoeae lineages.

The utility of the ResistancePlus GC assay in RGT depends on the relative frequency and locations of mutations across lineages of ciprofloxacin resistance mutations, particularly *gyrA* S91F. A collection of 8,179 nonduplicated (one isolate per individual) N. gonorrhoeae global isolates with available MIC data were obtained from Pathogenwatch (11), including isolates from Victoria, Australia (12). Of these, 3,144 isolates were phenotypically resistant to ciprofloxacin and were examined for mutations in *gyrA* and *parC.* Isolates were defined as resistant to ciprofloxacin if the MIC was ≥ 1 μg/ml as per Clinical and Laboratory Standards Institute (CLSI) guidelines (13) (Table S2). In total, 3,100/3,144 (98.6%) of isolates identified as phenotypically ciprofloxacin-resistant had the S91F mutation and 108/5,035 (2.1%) of isolates identified as phenotypically ciprofloxacin-susceptible had the S91F mutation. Accordingly, the sensitivity and specificity of the *gyrA* S91F mutation for conferring ciprofloxacin resistance in N. gonorrhoeae isolates was 98.6% and 97.9%, respectively. In addition, 3,095/3,100 (99.8%) of isolates with an S91F mutation also harbored a D95 mutation, most commonly D95G (1,996/3,095; 64.5%), D95A (954/3,095; 30.8%), or D95N (145/3,095; 4.7%). Further, mutations in *parC* were identified in 2,573/3,144 (81.8%) of ciprofloxacin-resistant N. gonorrhoeae. These included mutations at S87 (1,964/2,573; 76.3%), D86 (596/2,573; 23.2%), S88 (106/2,573; 4.1%), and E91 (49/2,573; 1.9%). Of the 44/3,144 (1.4%) ciprofloxacin-resistant N. gonorrhoeae isolates that did not have a *gyrA* S91F mutation, 2/44 (4.6%) carried a *parC* mutation (1: D86N, 1: S87R), with no other *gyrA* or *parC* mutations identified in the remaining isolates. Ciprofloxacin-resistant isolates with a *gyrA* S91F mutation were identified across 16 multilocus sequence types (STs) (Table S2), where the dominant STs were ST1901 (945/3,144; 30.1%) and ST7363 (425/3,144; 13.5%). One limitation of our approach was that we relied on phenotypic data reported by other studies, although we applied CLSI criteria for ciprofloxacin resistance to all isolates to enable a standardized comparison.

In summary, we evaluated the sensitivity and specificity for detection of N. gonorrhoeae using a commercially available ciprofloxacin RGT assay, with positive results more likely using samples with higher RLU values on the AC2 assay. The bacterial load of N. gonorrhoeae varies between anatomical sites and may therefore affect sensitivity of the assay (14). We also found that indeterminate *gyrA* results were more likely at lower RLUs from extragenital sites, possibly suggesting a lower bacterial load in these samples and/or potential cross-reactivity with nongonococcal *Neisseria* isolates. This “therapeutic gap” (i.e., positive for N. gonorrhoeae using TMA and negative for N. gonorrhoeae and/or *gyrA* indeterminate using the ResistancePlusGC assay) is likely due to differential analytical sensitivity between the TMA-based and PCR-based assays. Although this only constituted a minority of samples in our study (15.2%), in clinical practice this would mean a proportion of patients would have treatment with empirical rather than “tailored” therapy. Importantly, the clinical and economic utility of resistance-guided therapy for ciprofloxacin is likely to vary based on local rates of ciprofloxacin resistance. Finally, we found that the GyrA S91F mutation was both highly predictive for ciprofloxacin resistance and stable across a range of N. gonorrhoeae lineages from multiple geographic settings. Collectively, our work further supports the feasibility of implementing RGT for gonorrhea into routine molecular testing. Future work should explore improved integration of assays for RGT into large-scale NAAT workflows for gonorrhea and monitor clinical outcome data in patients treated using RGT.

## Supplementary Material

Supplemental file 1
