# Supplementary material for: Reflex Detection of Ciprofloxacin Resistance in Neisseria gonorrhoeae by Use of the SpeeDx ResistancePlus GC Assay
Source: J Clin Microbiol. 2021 Apr 20;59(5):e00089-21. doi: 10.1128/JCM.00089-21 (PMC8091848; doi:10.1128/JCM.00089-21)
Supplement: Supplemental file 1 [file JCM.00089-21-s0001.pdf]

SUPPLEMENTARY APPENDIX

Reflex detection of ciprofloxacin resistance in *Neisseria gonorrhoeae*

using the SpeeDx ResistancePlus® GC assay

Darren Y.J. Lee[1], Melinda M. Ashcroft[1], Eric P.F. Chow[2,3,4], Michelle Sait[5], Vesna De Petra[5], Marlene Tschaepe[5], Sigrid Lange[5], George Taiaroa[1], Catriona S Bradshaw[2,3], David M Whiley[6], Christopher K Fairley[2,3], Benjamin P. Howden[5], Marcus Y. Chen[2,3], Shivani Pasricha[1], Deborah A. Williamson[1,5,7*]

[1] *Department of Microbiology and Immunology, The Peter Doherty Institute for Infection and Immunity at The University of Melbourne, Melbourne, Victoria, Australia*

[2] *Melbourne Sexual Health Centre, Alfred Health, Carlton, Victoria, Australia*

[3] *Central Clinical School, Monash University, Melbourne, Victoria, Australia*

[4] *Centre for Epidemiology and Biostatistics, Melbourne School of Population and Global Health, The University of Melbourne, Melbourne, Victoria, Australia*

[5] *Microbiological Diagnostic Unit Public Health Laboratory, The Peter Doherty Institute for Infection and Immunity at The University of Melbourne, Melbourne, Victoria, Australia*

[6] *The University of Queensland Centre for Clinical Research (UQ-CCR), Faculty of Medicine, The University of Queensland, Brisbane, Queensland, Australia*

[7] *Department of Microbiology, Royal Melbourne Hospital, Melbourne, Victoria, Australia*

SUPPLEMENTARY METHODS

Bioinformatic analysis

The metadata table, typing table and the antimicrobial resistance (AMR) SNPs table from each *N. gonorrhoeae* collection on Pathogenwatch was downloaded and manually checked for ciprofloxacin Minimum Inhibitory Concentration (MIC) data (https://pathogen.watch/collections/all; downloaded on 28th Nov, 2020) (1). Only those collections that contained ciprofloxacin MIC data or were from our previous work (2) were kept for subsequent analysis. Isolates were defined as resistant to ciprofloxacin if the MIC was ≥ 1 µg/mL as per Clinical and Laboratory Standards Institute (CLSI) guidelines (3). Duplicate isolates (those that were present in multiple collections) were filtered so that only a single representative remained and all isolates without MIC data were removed from the analysis. Multi-locus Sequence Types within Pathogenwatch were determined based on the *Neisseria* scheme on PubMLST (4). Typing of AMR genes within Pathogenwatch is species-specific, with each database compiled from literature searches and publicly available databases such as CARD, ResFinder and NCBI (5). Only mutations previously associated with ciprofloxacin resistance in *gyrA* and *parC* were analysed.

Statistical analysis

Sensitivity and specificity were calculated based on data provided in Pathogenwatch. Sensitivity for the *gyrA* S91F mutation was calculated by dividing the proportion of isolates with the *gyrA* S91F mutation conferring ciprofloxacin resistance by the total number of isolates with phenotypic ciprofloxacin resistance. Specificity was calculated by dividing the proportion

of ciprofloxacin susceptible isolates without the *gyrA* S91F mutation by the total number of ciprofloxacin susceptible isolates.

SUPPLEMENTARY RESULTS

Table S1. Overall performance of the SpeeDx ResistancePlus® GC assay compared to the standard AC2 assay for the detection of *Neisseria gonorrhoeae* and *gyrA*.

AC2 assay	SpeeDx ResistancePlus® GC assay				
RLU values	*N. gonorrhoeae* detected, *gyrA* indeterminate	*N. gonorrhoeae* detected, *gyrA* mutation detected	*N. gonorrhoeae* detected, *gyrA* mutation not detected	*N. gonorrhoeae* not detected	Total
0 – 1,000	22 (5.5%)	2 (0.5%)	7 (1.8%)	18 (4.5%)	49
1,000 – 1,500	28 (7.0%)	40 (10.0%)	67 (16.8%)	2 (0.5%)	137
> 1,500	0	81 (20.2%)	132 (33.0%)	1 (0.2%)	214
Total	50	123	206	21	400

Table S2. Summary of *Neisseria gonorrhoeae* isolates available on Pathogenwatch (1) with Minimum Inhibitory Concentration data.

Collection	Major Country / Region	Total no. isolates	No. duplicates removed	No. isolates CIP MICs	No. isolates CIP^R	Major CIP^R MLSTs	CIP^R with *gyrA*_S91F	CIP^R without *gyrA*_S91F	CIP^R with *gyrA*_D95	CIP^R with *parC*	Citation
Alfsnes_2020	Norway	816	0	816	356	7363 (52/356); 1901 (51/356); 8143 (29/356); 1587 (27/356); 7827 (25/356)	352/356	4 (1 has parC_D86N)	174(A); 164(G); 14(N); 352(TOT)	124(D86N); 10(S87I); 31(S87N);111(S87R); 16(S88P);8(E91K); 72(S91FgyrA_no_parC)	(6)
EuroGASP_2013	EU	1054	0	720	332	1901 (135/332); 7363 (61/332); 1588 (29/332)	332/332	0	83(A); 238(G); 11(N); 332(TOT)	31(D86N); 8(S87I); 32(S87N); 187(S87R); 5(S88P); 2(E91K); 73(S91FgyrA_no_parC)	NA
Demczuk_2015	Canada	168	0	168	102	1901 (74/102)	100/102	2 (1 has parC_S87R)	2(A); 92(G); 4(N); 98(TOT)	6(D86N); 1(S87I); 88(S87R); 3(S88P); 6(S91FgyrA_no_parC)	(7)
Grad_2014	USA	216	0	216	158	1901 (133/158)	158/158	0	12(A); 145(G); 1(N); 158(TOT)	1(D86N); 2(S87I); 8(S87N); 144(S87R); 3(S91FgyrA_no_parC)	(8)
Grad_2016	USA	1035	215; Grad_2014	820	403	1901 (307/403); 7363 (20/403)	399/403	4	18(A); 378(G); 3(N); 399(TOT)	5(D86N); 2(S87I); 7(S87N); 360(S87R); 1(S88P); 1(E91K); 25(S91FgyrA_no_parC)	(9)
Kwong_2018	Australia	75	0	75	35	1901 (19/35); 7363 (5/35)	35/35	0	2(A); 33(G); 35(TOT)	6(D86N); 2(S87N); 20(S87R); 2(E91K); 7(S91FgyrA_no_parC)	(10)
Lee_2018	New Zealand	376	0	376	120	7363 (43/120); 1901 (20/120); 7365 (15/120)	118/120	2	32(A); 86(G); 118(TOT)	10(D86N); 1(S87I); 6(S87N); 36(S87R); 2(E91K); 65(S91FgyrA_no_parC)	(11)
Mortimer_2020	USA	891	0	869	209	1901 (80/209); 7363 (47/209); 1588 (31/209)	209/209	0	41(A); 168(G); 209(TOT)	6(D86N); 2(S87I); 26(S87N); 112(S87R); 4(E91K); 63(S91FgyrA_no_parC)	(12)
Osnes_2020	Norway	133	25; Alfsnes_2020	15	15	7827 (14/15)	15/15	0	15(G); 15(TOT)	15(D86N); 0(S91FgyrA_no_parC)	(13)
Schmerer_2020	USA	324	129; Thomas_2019	195	32	8143 (5/32); 9363 (5/32); 7371 (3/32); 7827 (3/32); 13526 (3/32); 1588 (3/32	29/32	3	15(A); 11(G); 3(N); 29(TOT)	11(D86N); 3(S87I); 3(S87N); 11(S87R); 1(E91K); 1(S91FgyrA_no_parC)	(14)
Sanchez-Buso_2019	Global	395	0	386	146	1901 (56/146); 1588 (18/146); 7363 (7/146)	145/146	1	32(A); 88(G); 23(N); 143(TOT)	14(D86N); 21(S87N); 72(S87R); 11(S88P); 11(E91K); 32(S91FgyrA_no_parC)	(15)
Thomas_2019	USA	644	0	129	32	9363 (9/32); 1901 (7/32); 7371 (4/32)	32/32	0	18(A); 10(G); 4(N); 32(TOT)	15(D86N); 4(S87I); 13(S87R); 0(S91FgyrA_no_parC)	(16)

Town_2020	UK	1288	0	1016	367	7822 (30/367); 7363 (25/367); 11428 (23/367); 8143 (20/367); 1579 (20/367)	340/367	27	172(A); 166(G); 1(N); 339(TOT)	61(D86N); 6(S87I); 23(S87N); 163(S87R); 1(E91K); 87(S91FgyrA_no_parC)	(17)
Williamson_2019	Australia	2179	0	2179	670	8143 (177/670); 7363 (133/670); 11981 (118/670); 1901 (32/670); 7827 (27/670)	670/670	0	344(A); 297(G); 29(N); 670(TOT)	241(D86N); 17(S87I); 63(S87N); 259(S87R); 26(S88P); 16(E91K); 93(S91FgyrA_no_parC)	(2)
Yahara_2018	Japan	245	0	199	167	7363 (32/167); 1901 (31/167); 1579 (26/167)	166/167	1	9(A); 105(G); 52(N); 166(TOT)	23(D86N); 5(S87N); 105(S87R); 44(S88P); 1(E91K); 1(S91FgyrA_no_parC)	(18)

EU = Europe, USA = United States of America; UK = United Kingdom; CIP^R = Ciprofloxacin-resistant; CIP^S = Ciprofloxacin-susceptible; MIC = Minimum Inhibitory Concentration; MLST = Multi-locus Sequence Type; TOT = total

REFERENCES

1. Sánchez-Busó L, Yeats CA, Taylor B, Goater R, Underwood A, Abudahab K, Argimón S, Ma KC, Mortimer TD, Cole MJ, Grad YH, Martin I, Raphael BH, Shafer WM, Spiteri G, Town K, Wi T, Harris SR, Unemo M, Aanensen DM. 2020. A community-driven resource for genomic surveillance of *Neisseria gonorrhoeae* at Pathogenwatch. *bioRxiv* doi:10.1101/2020.07.03.186726.
2. Williamson DA, Chow EPF, Gorrie CL, Seemann T, Ingle DJ, Higgins N, Easton M, Taiaroa G, Grad YH, Kwong JC, Fairley CK, Chen MY, Howden BP. 2019. Bridging of *Neisseria gonorrhoeae* lineages across sexual networks in the HIV pre-exposure prophylaxis era. *Nat Commun* 10:3988.
3. Clinical and Laboratory Standards Institute. 2020. M100 Performance Standards for Antimicrobial Susceptibility Testing. Wayne, PA, USA.
4. Jolley KA, Bray JE, Maiden MCJ. 2018. Open-access bacterial population genomics: BIGSdb software, the PubMLST.org website and their applications. *Wellcome Open Res* 3:124.
5. Pathogenwatch. 2019. Pathogenwatch AMR. https://cgps.gitbook.io/pathogenwatch/technical-descriptions/antimicrobial-resistance-prediction/pw-amr. Accessed 04/12/20.
6. Alfsnes K, Eldholm V, Olsen AO, Brynildsrud OB, Bohlin J, Steinbakk M, Caugant DA. 2020. Genomic epidemiology and population structure of *Neisseria gonorrhoeae* in Norway, 2016-2017. *Microb Genom* 6.
7. Demczuk W, Lynch T, Martin I, Van Domselaar G, Graham M, Bharat A, Allen V, Hoang L, Lefebvre B, Tyrrell G, Horsman G, Haldane D, Garceau R, Wylie J, Wong T, Mulvey MR. 2015. Whole-genome phylogenomic heterogeneity of *Neisseria gonorrhoeae* isolates with decreased cephalosporin susceptibility collected in Canada between 1989 and 2013. *J Clin Microbiol* 53:191-200.
8. Grad YH, Kirkcaldy RD, Trees D, Dordel J, Harris SR, Goldstein E, Weinstock H, Parkhill J, Hanage WP, Bentley S, Lipsitch M. 2014. Genomic epidemiology of *Neisseria gonorrhoeae* with reduced susceptibility to cefixime in the USA: a retrospective observational study. *Lancet Infect Dis* 14:220-6.
9. Grad YH, Harris SR, Kirkcaldy RD, Green AG, Marks DS, Bentley SD, Trees D, Lipsitch M. 2016. Genomic Epidemiology of Gonococcal Resistance to Extended-Spectrum Cephalosporins, Macrolides, and Fluoroquinolones in the United States, 2000-2013. *J Infect Dis* 214:1579-1587.
10. Kwong JC, Chow EPF, Stevens K, Stinear TP, Seemann T, Fairley CK, Chen MY, Howden BP. 2018. Whole-genome sequencing reveals transmission of gonococcal antibiotic resistance among men who have sex with men: an observational study. *Sex Transm Infect* 94:151-157.
11. Lee RS, Seemann T, Heffernan H, Kwong JC, Gonçalves da Silva A, Carter GP, Woodhouse R, Dyet KH, Bulach DM, Stinear TP, Howden BP, Williamson DA. 2018. Genomic epidemiology and antimicrobial resistance of *Neisseria gonorrhoeae* in New Zealand. *J Antimicrob Chemother* 73:353-364.
12. Mortimer TD, Pathela P, Crawley A, Rakeman JL, Lin Y, Harris SR, Blank S, Schillinger JA, Grad YH. 2020. The distribution and spread of susceptible and resistant *Neisseria gonorrhoeae* across demographic groups in a major metropolitan center. *medRxiv* doi:10.1101/2020.04.30.20086413.
13. Osnes MN, Didelot X, de Korne-Elenbaas J, Alfsnes K, Brynildsrud OB, Syversen G, Nilsen Ø, de Blasio BF, Caugant DA, Eldholm V. 2020. The sudden emergence of a

Neisseria gonorrhoeae strain with reduced susceptibility to extended-spectrum cephalosporins, Norway. *bioRxiv* doi:10.1101/2020.02.07.935825.

14. Schmerer MW, Abrams AJ, Seby S, Thomas JCt, Cartee J, Lucking S, Vidyaprakash E, Pham CD, Sharpe S, Pettus K, St Cyr SB, Torrone EA, Kersh EN, Gernert KM. 2020. Genomic Characterization of *Neisseria gonorrhoeae* Strains from 2016 U.S. Sentinel Surveillance Displaying Reduced Susceptibility to Azithromycin. *Antimicrob Agents Chemother* 64.
15. Sánchez-Busó L, Golparian D, Corander J, Grad YH, Ohnishi M, Flemming R, Parkhill J, Bentley SD, Unemo M, Harris SR. 2019. The impact of antimicrobials on gonococcal evolution. *Nat Microbiol* 4:1941-1950.
16. Thomas JC, Seby S, Abrams AJ, Cartee J, Lucking S, Vidyaprakash E, Schmerer M, Pham CD, Hong J, Torrone E, Cyr SS, Shafer WM, Bernstein K, Kersh EN, Gernert KM. 2019. Evidence of Recent Genomic Evolution in Gonococcal Strains With Decreased Susceptibility to Cephalosporins or Azithromycin in the United States, 2014-2016. *J Infect Dis* 220:294-305.
17. Town K, Field N, Harris SR, Sánchez-Busó L, Cole MJ, Pitt R, Fifer H, Mohammed H, Hughes G. 2020. Phylogenomic analysis of *Neisseria gonorrhoeae* transmission to assess sexual mixing and HIV transmission risk in England: a cross-sectional, observational, whole-genome sequencing study. *Lancet Infect Dis* 20:478-486.
18. Yahara K, Nakayama SI, Shimuta K, Lee KI, Morita M, Kawahata T, Kuroki T, Watanabe Y, Ohya H, Yasuda M, Deguchi T, Didelot X, Ohnishi M. 2018. Genomic surveillance of *Neisseria gonorrhoeae* to investigate the distribution and evolution of antimicrobial-resistance determinants and lineages. *Microb Genom* 4.
